# Role of peribrachial fat as a key determinant of brachial artery dilatation for successful arteriovenous fistula maturation in hemodialysis patients

**DOI:** 10.1038/s41598-020-60734-8

**Published:** 2020-03-02

**Authors:** Hyung Seok Lee, Mi Jin Park, Sam Youl Yoon, Narae Joo, Young Rim Song, Hyung Jik Kim, Sung Gyun Kim, Victor Nizet, Jwa-Kyung Kim

**Affiliations:** 1 Department of Internal Medicine & Kidney Research Institute, Hallym University Sacred Heart Hospital, Anyang, Korea; 2 Department of Clinical Immunology, Hallym University Sacred Heart Hospital, Anyang, Korea; 3 Division of General Surgery, Hallym University Sacred Heart Hospital, Anyang, South Korea; 4 Division of Host-Microbe Systems and Therapeutics, Department of Pediatrics, School of Medicine, University of California, San Diego, La Jolla, CA United States

**Keywords:** Kidney diseases, Metabolic disorders

## Abstract

The functional quality of the inflow artery is one of the most important determinants of arteriovenous fistula (AVF) success. We evaluated the association of early optimal brachial arterial dilatation with a successful AVF maturation and assessed the role of peribrachial adipose tissue in determining brachial arterial distensibility. All patients underwent a preoperative vascular mapping with Doppler ultrasound (US), and only patients who had suitable vessels for AVF creation were enrolled (n = 162). Peribrachial fat thickness was measured using US. To evaluate the degree of brachial dilatation, follow-up US was performed at 1 month after surgery, and early brachial artery dilation was defined as the change in postoperative arterial diameter compared to the preoperative value. The primary outcome was failure to achieve a clinically functional AVF within 8 weeks. Nonfunctional AVF occurred in 21 (13.0%) patients, and they had a significantly lower brachial dilatation than patients with successful AVF during early period after surgery (0.85 vs. 0.43 mm, p = 0.003). Patients with a brachial dilatation greater than median level showed a 1.8-times higher rate of achieving a successful AVF than those without. Interestingly, the early brachial dilatation showed significant correlations with diabetes (r = −0.260, p = 0.001), peribrachial fat thickness (r = −0.301, p = 0.008), and the presence of brachial artery calcification (r = −0.178, p = 0.036). Even after adjustments for demographic factors, comorbidities, and baseline brachial flow volume, peribrachial fat thickness was an independent determinant for early brachial dilatation (β = −0.286, p = 0.013). A close interplay between the peri-brachial fat and brachial dilatation can be translated into novel clinical tools to predict successful AVF maturation.

## Introduction

Arteriovenous (AV) fistula (AVF) is a preferred type of vascular access for hemodialysis (HD), because it is associated with fewer complications, improved access survival, and lower risk of patient mortality compared to AV graft or central venous catheter^1^. Even in elderly patients, AVF remains the best mode of HD^2^. However, maturation failure has been described as a major limitation of its use. To minimize AVF maturation failure, the rule of thumb is a judicious selection of adequate vessels and early detection of fistula complications. Doppler ultrasound (US) is widely available for preoperative vascular mapping; the minimum requirement for successful AVF creation is an arterial diameter of greater than 2.0 mm^3–6^. However, AVF success rates as low as 60% are reported, even with arterial diameters above 2.0 mm^7^. Therefore, we have to focus on the functional quality of vessels such as arterial blood flow or the artery’s ability to dilate rather than indicating a threshold for the diameter of the vessels^8,9^. Arterial distensibility is not necessarily related to the internal diameter and could be an important determinant of AVF success.

Adequate fistula maturation might be closely associated with an optimal dilation of the brachial artery after fistula creation^10^. The brachial artery is usually the main feeding artery of the AVF, either directly (upper arm AVF), or through the radial artery (forearm AVF). The degree of brachial arterial dilatation and resultant increase in brachial flow volume (FV) could provide valuable information for the early prediction of AVF maturation failure beyond the traditional predictors. Previous studies on functional Doppler parameters such as flow-mediated dilation (FMD) or nitroglycerin-mediated dilation (NMD) have suggested that the ability of the artery to dilate plays a significant role in AVF maturation or functional patency^8,11–13^.

Recent studies implicate perivascular adipose tissue (PVAT), which surrounds the artery wall, as playing an important role in the pathogenesis of vascular disease^14–16^. PVAT serves not only as a structural support for most arteries but also as an active component of the vessel wall^17^. Adipokines and inflammatory cytokines released from the PVAT can diffuse into the vascular wall in a paracrine manner, directly affecting vascular biology, structure, and function^16,18^. Since it is adjacent to blood vessels, dysfunctional PVAT may have more pronounced effects on vascular function than other adipose tissue depots. There are considerable evidences to demonstrate the close association between pericardial fat, defined as fat immediately surrounding the coronary arteries, and coronary plaques as well as the development and progression of coronary artery disease (CAD)^19,20^. All the studies suggested a local effect of pericardial fat on coronary inflammation and resultant progression of atherosclerosis^21–23^. In addition, a recently published data showed that baseline phenotypic characteristics of the adipose tissue juxtaposed to the developing AVF strongly associated with venous remodeling after surgery^24^.

The aim of this study was to evaluate the association of early optimal brachial arterial dilatation with the successful AVF maturation in newly created AVFs. In addition, we previously reported that higher levels of serum leptin are closely associated with pre-existing vasculopathy and resultant higher rate of AVF maturation failure in HD patients^25^. Considering that leptin is a major adipokine from adipose tissue, we evaluated the effect of peribrachial PVAT on postoperative dilatation of the brachial artery, too.

## Results

### Patient characteristics and vascular outcomes

A total of 162 HD patients with newly created AVFs (upper arm, 71 [43.8%]; forearm, 91 [56.2%]) were analyzed. None underwent a two-stage transposition fistula in upper arm AVF. The mean age was 64.1 ± 12.1 years; 49 (30.2%) and 13 (8.0%) patients were over 70 and 80 years, respectively. Diabetes was the most common cause of end stage renal disease (ESRD) (60.5%). All patients had a preoperative vascular mapping with Doppler US, and postoperative follow-up US was performed at 32.3 ± 12.1 days after operation.

The mean duration until the first use of the AVF was 54.9 ± 21.2 days. As shown in Table 1, the failure to achieve a clinically functional AVF within 8 weeks occurred in 21 (13.0%) cases; 18 cases were primary maturation failure and 3 cases were inability to cannulate the AVF due to edema, pain, infiltration, or tortuosity. The incidence of primary maturation failure was much lower than that of previous reports, in which the failure rate reached 46%^25^. Vascular interventions before the first needling of the AVF were done in 16 (9.8%) patients within a mean of 51.1 ± 18.1 days after operation (11 cases with percutaneous transluminal angioplasty [PTA], 4 cases with accessory vein ligation [AVL] and 1 case acute thrombus removal).

**Table 1:** Baseline characteristics and vascular outcomes of patients who newly created AVFs.

Variables	Total patients (n = 162)	Clinically functional AVFs until 8 weeks		
		Success (n = 141, 87.0%)	Failure (n = 21, 13.0%)	P
Age (years)	64.1 ± 12.1	64.1 ± 11.1	65.5 ± 13.9	0.642
Gender, male, n (%)	98 (60.5)	87 (61.7)	11 (52.4)	0.280
BMI, kg/m^2^	25.1 ± 4.3	24.8 ± 3.7	25.4 ± 4.7	0.624
Diabetes, n (%)	103 (64.0)	89 (63.6)	14 (66.7)	0.494
Previous CAD, n (%)	34 (20.9)	27 (19.1)	7 (33.3)	0.177
Cause of ESRD, n (%)				0.405
Diabetic	98 (60.5)	84 (59.6)	14 (66.7)	
Hypertensive	37 (22.8)	33 (23.4)	4 (19.0)	
Glomerulonephritis	11 (6.8)	9 (6.4)	2 (9.5)	
Others	16 (9.8)	15 (9.9)	1 (4.8)	
Systolic blood pressure (mmHg)	145.1 ± 19.9	146.1 ± 19.9	140.6 ± 19.0	0.336
Diastolic blood pressure (mmHg)	76.5 ± 12.1	77.7 ± 12.7	72.2 ± 13.0	0.101
Types				0.212
Forearm AVFs, n (%)	91 (56.2)	77 (84.6)	14 (15.4)	
Upper arm AVFs, n (%)	71 (43.8)	64 (90.1)	7 (9.9)	
**Biochemical parameters**				
WBC, cells/mm^3^	6,812 ± 1,870	6,924 ± 1,840	6,510 ± 2,010	0.450
Hemoglobin (g/dL)	9.4 ± 1.6	9.3 ± 1.4	9.8 ± 2.4	0.312
Neutrophil, cells/mm^3^	4,708 ± 1,716	4,115 ± 1,814	4,852 ± 1,120	0.138
N/L ratio	4.2 ± 2.5	3.6 ± 2.1	4.5 ± 2.3	0.150
BUN, mg/dL	80.8 ± 30.2	79.3 ± 30.3	86.5 ± 29.2	0.402
Creatinine, mg/dL	7.6 ± 3.1	7.6 ± 3.3	7.6 ± 2.4	0.952
Albumin, g/dL	3.5 ± 0.5	3.5 ± 0.5	3.5 ± 0.5	0.957
Total cholesterol, mg/dL	152.7 ± 47.5	151.1 ± 50.4	156.0 ± 35.7	0.719
**Body composition analysis**				
Lean Tissue Index	12.7 ± 5.3	12.4 ± 4.2	11.6 ± 5.0	0.353
Fat Tissue Index	11.6 ± 5.0	11.4 ± 4.7	13.0 ± 6.5	0.203
Percentage of body fat (%)				
female	29.3 ± 10.3	29.3 ± 10.1	29.6 ± 12.7	0.930
male	37.3 ± 9.8	37.1 ± 9.2	38.1 ± 13.2	0.787
ECW/ICW ratio	1.0 ± 0.2	1.0 ± 0.2	1.0 ± 0.2	0.924
Overhydration, L	2.2 ± 2.9	2.3 ± 2.9	1.6 ± 2.8	0.360

All data are expressed as mean ± SD. Abbreviations: BMI, body mass index; CAD, coronary artery disease; ESRD, end-stage renal disease; BUN, blood urea nitrogen; ECW, extracellular water; ICW, intracellular water.

Since only patients who had suitable vessels for making AVFs were enrolled, there was no difference in age, gender, BMI, diabetes, or other comorbidities between patients with or without a primary endpoint. Thus, the sole variables determining the primary outcomes were postoperative changes of the vessels (Table 1).

### Changes in diameter of vessels and primary outcomes

**Figure 1:**
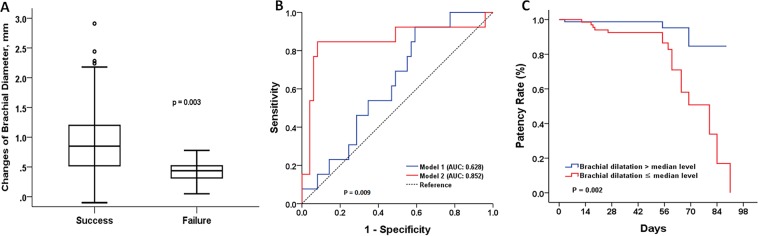
The role of early brachial dilatation for outcome prediction in hemodialysis patients. Comparison of brachial dilatation at 1-month after surgery between patients with or without maturation. (**A**) When early brachial dilatation was added to the predicting model, the AUC increased significantly. (**B**) Kaplan-Meier estimates for the patency rate. Patients with a brachial dilatation greater than 0.75 mm showed a 1.8-times higher rate of achieving a successful AVF than those without (**C**).

Table 2 summarizes the preoperative mapping data, postoperative follow-up findings, and changes in the early brachial arterial parameters, with respect to the primary outcomes. Because we selected suitable patients for AVF creation, the preoperative diameters of the radial arteries and cephalic veins are comparable between the two groups. However, the differences in the brachial arterial parameters are remarkable: at baseline, patients with a nonfunctional AVF had significantly lower brachial FV and a higher prevalence of brachial calcification than those with a successful AVF. At the follow-up examination, patients in the success group exhibited much greater brachial diameters, which accounts for the much greater postoperative brachial FV in this group. Accordingly, early brachial arterial dilatation at 1 month after surgery was much more prominent in the success group than in the failed group; the median change in brachial diameter was 0.85 (interquartile range [IQR], 0.52–1.22) vs. 0.43 (IQR, 0.30–0.52) mm, respectively (Fig. 1A).

**Table 2:** Comparisons of pre- and 1-month postoperative US findings according to primary endpoint.

Clinical characteristics	Failure of clinically functional AVFs until 8 weeks		
	Success (141, 87.0%)	Failure (21, 13.0%)	P
**Preoperative US**			
Brachial artery			
Diameter, mm	4.18 ± 0.67	3.90 ± 0.61	0.082
Peak systolic velocity, cm/s	66.4 ± 19.4	60.3 ± 15.1	0.210
Mean Velocity, cm/s	9.6 ± 4.4	9.9 ± 4.9	0.791
Flow Volume, mL/min	74.4 ± 37.3	52.0 ± 30.5	0.016
Calcification, n (%)	5 (3.5)	6 (28.5)	0.005
Radial artery, mm			
Diameter, mm	2.1 ± 0.3	2.0 ± 0.4	0.208
Calcification, n (%)	9 (6.3)	3 (14.2)	0.347
Cephalic vein, mm	2.9 ± 0.7	2.8 ± 0.5	0.542
**Postoperative US, 1month**			
Brachial artery			
Diameter, mm	5.07 ± 0.71	4.50 ± 0.65	0.001
Peak systolic velocity, cm/s	169.8 ± 67.1	83.9 ± 54.5	0.035
Mean Velocity, cm/s	109.5 ± 49.1	61.4 ± 47.6	0.009
Flow Volume, mL/min	1300.2 ± 594.2	328.7 ± 225.8	<0.001
RI	0.51 ± 0.09	0.86 ± 0.17	0.033
**Changes of brachial artery**			
Δ Diameter, median with IQR	0.85 (0.52–1.22)	0.43 (0.30–0.52)	0.003
Δ Diameter, %	24.5 ± 17.7	13.8 ± 11.4	0.011
Δ Flow volume, mL	1235.7 ± 582.9	283.2 ± 179.6	<0.001

In fact, an early brachial dilatation was a significant determinant for the successful AVF. Figure 1B showed the prediction model for the primary outcomes. In baseline model 1, which included age, gender, comorbidities (diabetes, hypertension and CAD), BMI, systolic blood pressure (BP), location of AVFs, and baseline brachial diameter, the AUC was 0.628. However, in the fully adjusted Model 2, which includes the addition of the degree of brachial dilatation, the AUC significantly increased to 0.852 (p = 0.009). Patients with a brachial dilatation greater than the median level of 0.75 mm had 1.8-times higher rate of achieving a successful AVF than those without (hazard ratio: 1.78, 95% CI: 1.24–2.51; p = 0.002) (Fig. 1C).

### Factors affecting brachial dilatation

**Figure 2:**
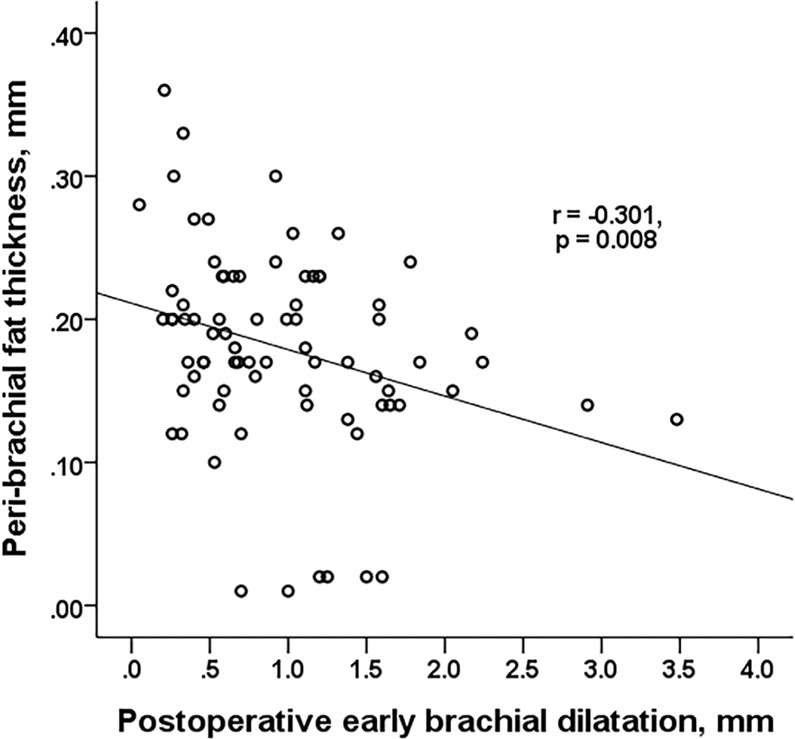
The peribrachial fat thickness had a significantly negative asociation with an early brachial dilatation after AVF creation.

The degree of early brachial dilatation after AVF creation had a negative correlation with diabetes (r = −0.275, p = 0.001), the presence of brachial calcification (r = −0.178, p = 0.036) and peribrachial fat thickness (r = −0.301, p = 0.008) (Fig. 2). While there was no detectable correlation with age, gender, or other demographic factors, upper AVF showed a close relationship with higher brachial dilatation, too (r = 0.181, p = 0.027) (Table 3).

**Table 3:** Correlation analysis.

	∆ Brachial diameter		Peri-brachial fat thickness		Brachial calcification	
	r	p	r	p		
Age	−0.129	0.139	0.158	0.213	−0.292	0.022
Gender	0.140	0.096	0.035	0.788	−0.051	0.569
DM	−0.260	0.001	0.215	0.062	0.113	0.183
Hypertension	−0.049	0.554	0.260	0.022	0.094	0.268
CAD	0.059	0.472	0.234	0.040	0.070	0.412
Systolic BP	−0.070	0.569	0.134	0.480	−0.302	0.809
Diastolic BP	0.118	0.324	0.140	0.462	−0.127	0.335
BMI	0.040	0.704	0.231	0.076	−0.172	0.205
AVF location	0.181	0.027	−0.091	0.432	0.139	0.101
LTI	−0.153	0.127	0.069	0.625	−0.182	0.068
FTI	0.032	0.740	0.031	0.830	0.100	0.319
OH	−0.115	0.247	−0.047	0.739	−0.002	0.988
Initial FV	−0.065	0.470	0.039	0.744	−0.051	0.569
Brachial calcification	−0.178	0.036	0.200	0.085	—	—
Peri-brachial fat thickness	−0.301	0.008	—	—	0.200	0.085
∆ Brachial diameter	—	—	−0.301	0.008	−0.178	0.036
∆ Brachial FV	0.278	0.003	0.125	0.778	0.181	0.627

Abbreviations: DM, diabetes mellitus; BMI, body mass index; LTI, lean tissue index; FTI, fat tissue index; OH, overhydration; FV, flow volume.

**Figure 3:**
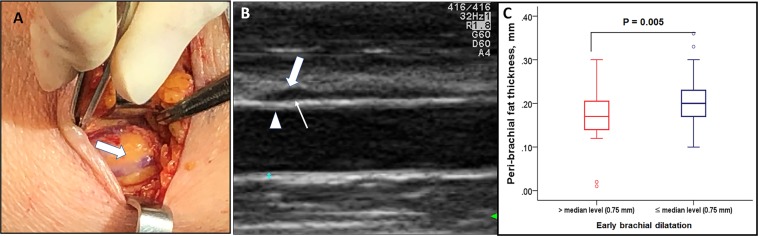
Representative imagings for peribrachial fat tissue and its association with brachial dilatation. Gross imaging at the time of surgery showed diffuse irregular fat deposit along the brachial artery (**A**) and it can be visualized in preoperative vascular mapping with US (**B**) Patients with brachial dilatation less than median level had a significantly thicker peribrachial fat (**C**).

The mean peribrachial fat thickness in ESRD patients was 0.18 ± 0.06 mm. Representative peribrachial fat findings are given in Fig. 3A,B. Peribrachial fat is located just outside the adventitia, and no fascial plane is observed between the adventitia and the surrounding adipose tissue. Patients with a postoperative brachial dilatation less than 0.75 mm (median level) had significantly thicker peribrachial fat than those with brachial dilatation greater than the median level (Fig. 3C). Interestingly, there was no significant correlation between peribrachial fat thickness and biochemical or body composition data, including fat mass (although there was a weak association with BMI). However, it showed a significant relationship with hypertension and previous CAD history.

Stepwise multiple regression analysis was performed to examine the independent roles of peribrachial fat thickness and brachial calcification in the determination of postoperative brachial dilatation. As shown in Table 4, both parameters were significant factors for adequate brachial dilatation in the univariate analysis. After adjustments for demographic factors (Model 1) and baseline brachial FV and AVF location (Model 2), both markers seemed to be independent determinants for adequate brachial dilatation. Even after adjusting for comorbidities that could affect vascular function, such as diabetes, hypertension, and CAD history (Model 3), the close relationship between peribrachial fat thickness and postoperative brachial dilatation was consistently observed (β = −0.286, p = 0.013). Notably, the role of peribrachial fat thickness for determining brachial dilatation was independent to the presence of brachial calcification, too (β = −0.240, p = 0.041).

**Table 4:** Univariate and multivariate linear regression analysis: role of peri-brachial fat thickness and brachial calcification on brachial dilatation.

∆ Brachial diameter	Univariate analysis		Multivariate analysis					
			Model 1		Model 2		Model 3	
	β	P	β	P	β	P	β	P
Peri-brachial fat	−0.301	0.008	−0.316	0.009	−0.304	0.012	−0.286	0.013
Brachial calcification	−0.178	0.036	−0.195	0.027	−0.236	0.010	−0.218	0.014

β, standardized regression coefficient.

Model 1, adjusted for age and gender.

Model 2, model 1 + adjustment for baseline brachial arterial FV and AVF location.

Model 3, model 2 + adjustment for comorbidities (diabetes, hypertension and CAD).

## Discussion

The main findings of this study are: (1) an optimal early brachial arterial dilatation with concomitant increase in brachial FV a key factor associated with a functional successful AVF; even though vessel diameter is adequate, an insufficient postoperative brachial dilatation associates with AVF maturation failure. (2) the degree of early brachial arterial dilatation had a close negative association with diabetes, brachial calcification, and peribrachial fat thickness; and (3) peribrachial fat is a major determinant for adequate brachial dilatation independent of traditional vascular risk factors or baseline brachial FV.

With the current practice guideline, which emphasizes the placement of an AVF, many efforts have been devoted to reduce the AVF maturation failure rate^26^. Until now, the best way to avoid a nonfunctional AV access was through the precise selection of vessels; this is why Doppler US is widely used for preoperative vascular mapping^27^. However, it can also provide valuable information on the health of the arterial system, including arterial diameter, wall thickness, course, calcifications, and PVAT thickness^8,28^. To date, the most commonly used Doppler US criteria for selecting vessels has been the diameter of the radial artery and cephalic vein. Although the likelihood of AVF patency and survival increases with the diameter of the vessels used to create the AVF, another important determinant of AVF success is functional quality of the arteries, which is not necessarily related to the internal diameter of the arteries^13^. In this regard, an adequate fistula maturation after surgery is associated with optimal dilation of the artery that feeds the AVF. Because the brachial artery is usually the main feeding artery of an AVF, we focused on the relationship between an optimal early brachial arterial dilatation and AVF success. To our knowledge, there is little data available regarding this issue, although its importance is widely recognized.

A strength of our study is that we selected only suitable patients for AVF creation, so baseline demographic parameters, as well as vessel diameters, were similar between the patients exhibiting successful maturation and those exhibiting maturation failure. However, brachial parameters were dissimilar between the two groups: not only was the baseline brachial FV lower in the maturation failure group, but brachial dilation after surgery was also significantly less than that in the successful group. Thus, we conclude that the degree of early brachial dilatation is a key factor for successful AVF maturation. Patients with a brachial dilatation greater than 0.75 mm within 1 month of surgery were twice as likely to achieve a successful AVF as those with a brachial dilatation less than 0.75 mm. The most plausible explanation for this finding is that the degree of brachial change during early post-operation reflects the overall brachial arterial health, which may include endothelial function, wall abnormality, calcification, and even peribrachial adipose tissue. Any of these factors may be associated with brachial distensibility, so inadequate brachial dilatation may be causally related to failure of functional AV access. Similarly, the usefulness of brachial artery FMD as a predictor of long-term cardiovascular events as well as AVF maturation or functional patency is well-established^13,29^. Although the artery’s distensibility can be estimated preoperatively on the basis of variations in the radial artery Doppler spectrum during reactive hyperemia or FMD, our study focused on changes in the brachial artery, and therefore we did not apply the reactive hyperemia^8,9^.

Our statistical analyses indicated a close relationship between brachial dilatation and diabetes, brachial calcification, and peribrachial fat thickness. Indeed, diabetes and brachial calcification are well-known risk factors for atherosclerosis, but the association with peribrachial fat thickness is novel. Most arteries are surrounded by PVAT. Recent studies have shown that PVAT does not simply serve as a structural support for vasculature, but rather, as a metabolically active endocrine organ, it actually influences the vascular tone and diameter. PVAT can have local pathologic effects by direct compression of the vessels or by releasing numerous vasoactive factors including inflammatory adipocytokines^15^. As these adipocytokines diffuse through the adventitia and media to reach the endothelium and lumen, the PVAT could induce a potential ‘outside-in’ inflammatory cascade and affect vascular endothelial dysfunction and smooth muscle migration^30^. This theory is supported by the lack of a fascial plane between the adventitia and surrounding adipose tissue, as well as by the extension of the vasa vasorum into perivascular adipose tissue^31,32^. In addition, immunostaining of atherosclerotic human aortas has shown the presence of inflammatory cells at the junction of perivascular adipose tissue and the vascular adventitia. To date, many clinical studies have demonstrated that increased accumulation of epicardial adipose tissue/pericoronary adipose tissue, which surrounds coronary arteries, is associated with CAD as well as development and progression of atherosclerosis^20,21^. In contrast, very few studies have been performed on PVAT surrounding the brachial artery. However, it seems to represent a novel and important adipose tissue for the regulation of muscular blood flow, independent of other fat depots. To our knowledge, this study is the first to demonstrate the close association between peribrachial adipose tissue and the degree of postoperative brachial dilatation.

In general, the amount of PVAT increases with advanced adiposity^33,34^. Although our analyses showed a weak positive association between BMI and peribrachial fat thickness, they showed no significant association between fat mass or body fat composition and peribrachial fat thickness. Thus, we can draw no conclusion about the correlation between perivascular fat thickness and systemic adiposity in this study.

Several shortcomings of this study should be addressed. First, we focused on the functional quality of the brachial artery and evaluated pre- and post-operative changes in the brachial diameter. We did not use brachial FMD, which may yield more accurate results. As an indicator of endothelial dysfunction, FMD may provide more reliable data supporting the functional quality of early brachial dilatation. Second, although we speculate that peribrachial fat tissue inhibits adequate early vasodilation through the local release of adipocytokines, we did not measure the serum levels of adipocytokines. However, in an earlier study, we found that higher serum leptin levels were closely associated with pre-existing vasculopathy and concomitant higher rates of AVF maturation failure in HD patients^25^. Finally, as the number of patients was relatively small, we could not analyze separately the data obtained from upper arm AVFs versus forearm AVFs. Rather, we focused on the role of the brachial artery as a feeding artery, regardless of the location of the AVF.

We conclude: (1) optimal early brachial dilatation appears to be a key factor of a successful AVF; (2) short-term measurement of brachial arterial diameter using Doppler US is an effective method to estimate the degree of brachial dilatation; and (3) peribrachial fat thickness, which can be measured during preoperative vascular mapping with Doppler US, appears to influence the early brachial dilatation beyond the traditional risk factors including baseline vessel parameters.

## Materials and Methods

### Study population and sampling

This study included HD patients with newly made long-term vascular access at Hallym University Sacred Heart Hospital between January 2016 and December 2018. Of the 332 patients during this period, a total of 170 were excluded due to: (1) creation of an AV graft (n = 123), (2) inability to make a vascular access (n = 19), or (3) refusal to participate in our pre-/post-vascular surveillance program (n = 28). Therefore, 162 patients were included. None of the patients had a prior AVF in the same arm. The baseline demographic data obtained included age, sex, comorbidities, and clinical data regarding the underlying cause of renal disease. Baseline BMI was calculated as body weight/(height/100)^2^. Venous sampling was performed immediately prior to each patient’s mid-week HD session. Biochemical analyses of white blood cells, neutrophils, lymphocytes, platelets, and levels of hemoglobin, serum albumin, lipid profiles, urea nitrogen, creatinine, and uric acid were measured. The neutrophil-to-lymphocyte ratio was also calculated. At the start of HD, we routinely performed body composition analysis to obtain each patient’s volume status and body composition data using a portable whole-body bioimpedance spectroscopy device (Body Composition Monitor, Fresenius Medical Care, Bad Homburg, Germany). Objective indicators of fluid status, including estimates of overhydration, intracellular water, extracellular water, and total body water, as well as a fat tissue index, percentage of body fat, and lean tissue index, were acquired. We did not obtain informed consent, because preoperative vascular mapping using US is a widely recommended examination, and this study is a retrospective analysis of prospectively obtained data. Waiver of consent was approved by Hallym University Sacred Heart Hospital Institutional Review Board and conducted in accordance with the Declaration of Helsinki. This study was approved by Hallym University Sacred Heart Hospital Institutional Review Board.

### Preoperative vascular mapping and postoperative monitoring of AVF

A preoperative inspection and palpation of the superficial veins over the forearm were performed carefully by experienced nephrologists and surgeon. Pulsations of brachial, radial, and ulnar artery were palpated, and an Allen test was performed to detect the risk of distal ischemia. Preoperative vascular mappings were performed routinely using a duplex Doppler ultrasound (US). Duplex US was performed by experienced registered technologist (RVT) from our vascular clinic using 10–18 MHz linear transducers (ProSound F37, Hitachi Aloka Healthcare, Japan). Vessel diameters, courses, anatomical variation, depth of vein, and presence of any vascular abnormalities, such as plaques, stenosis, or diversions, were recorded. For brachial arteries, peak systolic velocities, FV, and resistive index were measured at 4–5 cm proximal to the elbow joint. Also, we measured the peribrachial fat at the thickest point of peribrachial fat tissue at the 4–5 cm proximal to the elbow joint. The presence of arterial calcification was also evaluated in the baseline US examinations.

We also performed postoperative vascular monitoring/surveillance to determine the degree of early brachial dilatation and any complications that could reduce AVF survival. It was routinely performed between 4–6 weeks after the creation to assess whether AVF maturation was achieved. AVF maturation was defined as a draining vein diameter greater than 4 mm and a brachial arterial flow rate greater than 400 mL/min^26,35^.

### Surgical procedures

One vascular surgeon performed all of the AVF creations by end-to-side anastomosis, preserving the perivascular subcutaneous tissue, including perivascular adipose tissue, under local anesthesia with 2% lidocaine.

### Primary outcome

According to the Clinical Trial Endpoints for Dialysis Vascular Access Project of the American Society of Nephrology Kidney Health Initiative, the primary endpoint of this study was a failure to achieve a clinically functional AV access (stage 3 physiologic maturation of AVF) by 8 weeks after creation^35^. The causes of nonfunctional AV access included: 1) primary unassisted maturation failure, which is defined as a failure to achieve adequate fistula size ≥ 0.4 cm and minimal blood flow ≥ 400 mL/min by an objective demonstration using duplex US by 4–8 weeks; and 2) cannulation-associated failure, which is an inability to cannulate AVF due to arm edema, pain, difficulty in hemostasis, deep location, or tortuosity. With postoperative vascular surveillance, prompt vascular interventions, such as PTA or AVL, were performed if any AVF complications were detected, as recommended in the Kidney Disease Outcomes Quality Initiative guidelines. Use of the AVFs was permitted after 6–8 weeks, after confirming maturity by US, and after two trained nurses first cannulated the AVFs.

### Statistical analysis

Variables with normal distributions were expressed as the mean ± standard deviation, while categorical variables were expressed as percentages and compared using the chi-square test. For skewed data, we used median with IQR. We tested correlations between continuous variables with Pearson’s r or Spearman’s rank correlation coefficient, as appropriate. Also, we tested the association between the perivascular fat thickness, brachial vascular calcification, and changes of brachial artery diameter using multivariable linear regression models. To show the relationship of changes in brachial diameter with the successful AVF, a receiver operating characteristics curve was constructed, and the areas under the curve (AUC) were compared using the MedCalc program. Model 1 consisted of age, gender, comorbidities, BMI, systolic BP, location of AVFs, and the baseline brachial diameter, while Model 2 incorporated the degree of brachial dilatation into Model 1. By multiple regression analysis, the effects of peribrachial fat and brachial calcification on the changes of brachial artery diameter were determined. Cumulative survival curves were derived using the Kaplan–Meier method, and differences between survival curves were compared using a log-rank test. A Cox proportional hazards model was used to identify independent factors for the development of endpoints. The predictive value was expressed as a hazard ratio with corresponding 95% confidence intervals [CIs]. P < 0.05 was accepted as significant. All statistical analyses were performed using SPSS version 20.0 (SPSS, Inc., Chicago, IL, USA).

## Data Availability

All data generated or analyzed during this study are included in this published article.
